# Uterine Artery Pulsatility Index in Singleton Pregnancies Conceived via Assisted Reproductive Technology Versus Spontaneous Conception: A Systematic Review and Meta-Analysis

**DOI:** 10.3390/diagnostics15172192

**Published:** 2025-08-29

**Authors:** Antonios Siargkas, Ioannis Tsakiridis, Areti Faka, Dimitra Kappou, Ioannis Papastefanou, Themistoklis Dagklis

**Affiliations:** 1 Third Department of Obstetrics and Gynecology, School of Medicine, Faculty of Health Sciences, Aristotle University of Thessaloniki, 54124 Thessaloniki, Greece; antonis.siargkas@gmail.com (A.S.); jdpap2@yahoo.co.uk (I.P.);; 2 Institute of Life, IASO General Hospital, 15123 Athens, Greece; d.kappou@yahoo.com; 3 Fetal Medicine Clinic, Monis Petraki 4, Kolonaki, 11521 Athens, Greece; 4 Department of Women and Children’s Health, Faculty of Life Sciences and Medicine, King’s College London, London WC2R 2LS, UK

**Keywords:** assisted reproductive technology, embryo transfer, in vitro fertilization, intracytoplasmic sperm injection, intrauterine insemination, oocyte donation, ovulation induction, placental function, preeclampsia, small for gestational age, uterine artery pulsatility index

## Abstract

**Background/Objectives:** Uterine artery pulsatility index (UtA-PI) is a key biomarker of placental function, but its clinical interpretation in assisted reproductive technology (ART) pregnancies is uncertain. This meta-analysis aimed to assess trimester- and method-specific UtA-PI differences between ART and spontaneous conceptions (SC) and to examine associated risks for preeclampsia (PE) and small-for-gestational-age (SGA) neonates to contextualize its findings. **Methods:** A systematic search of MEDLINE, Scopus, and the Cochrane Library was conducted through 25 June 2025. We included observational studies comparing UtA-PI and perinatal outcomes in singleton ART versus SC pregnancies. The primary outcome was the standardized mean difference (SMD) in first (until the 13^+6^ gestational week) and second trimester (14^+0^–23^+6^ gestational weeks) UtA-PI measurements; secondary outcomes were PE and SGA rates. Analyses were stratified by ART modalities. Random-effects models were used, and study quality was evaluated using the Newcastle–Ottawa Scale and risk of bias with QUIPS tool (INPLASY registration: INPLASY202560104). **Results:** Thirteen cohort studies were included. Overall, ART pregnancies had significantly lower UtA-PI values than SC in both the first (SMD = −0.28; 95% CI: −0.53 to −0.03) and second trimesters (SMD = −0.20; 95% CI: −0.36 to −0.04). These reductions were driven by oocyte donation (first-trimester SMD = −0.70; 95% CI: −1.21 to −0.18; second-trimester SMD = −0.46; 95% CI: −0.65 to −0.26) and artificial cycle frozen embryo transfers (ET) (first-trimester SMD = −0.69; 95% CI: −1.00 to −0.39). These lower UtA-PI values typically suggest better placental perfusion and a lower risk of placental related complications. However, ART pregnancies had an elevated overall risk for PE (risk ratio [RR] = 2.32; 95% CI: 1.72 to 3.12), with the highest risk in oocyte donation (RR = 6.11; 95% CI: 3.35 to 11.17) and artificial cycle frozen ET (RR = 3.45; 95% CI: 1.51 to 7.90). **Conclusions:** ART pregnancies, particularly from oocyte donation and artificial cycle frozen ET, show lower UtA-PI values despite a significantly elevated risk for PE. This finding suggests that mechanisms other than placental perfusion contribute to PE development. Clinically, the ART method is an independent risk factor for PE, and UtA-PI interpretation should be adjusted accordingly. Further research is crucial to elucidate the underlying pathophysiology.

## 1. Introduction

The evaluation of uterine artery blood flow, particularly through the measurement of the uterine artery pulsatility index (UtA-PI) via Doppler ultrasound, has become a fundamental tool in assessing placental vascular resistance during pregnancy [1,2]. As a non-invasive and reproducible technique, UtA-PI provides an indirect indicator of placental function, offering critical insight into fetal growth potential and the maternal cardiovascular adaptations that underpin successful gestation [3]. Notably, elevated UtA-PI values correlate with impaired placentation, a common underlying factor in several placental-related pregnancy complications, such as preeclampsia (PE) and small-for-gestational age (SGA) neonates [4,5,6]. The pathophysiological basis of this association lies in deficient trophoblastic invasion and insufficient remodeling of the maternal spiral arteries, which result in increased vascular resistance and compromised uteroplacental perfusion, ultimately reducing the delivery of oxygen and nutrients to the fetus [7].

PE is a major global health concern, ranking among the leading causes of maternal morbidity and mortality [8]. As such, there has been an increasing effort on early identification and prevention. In this context, UtA-PI is a key variable in first-trimester screening algorithms developed to estimate the risk of PE [9]. These predictive algorithms inform early interventions, such as the prescription of low-dose aspirin for women identified as high-risk, an approach that has demonstrated effectiveness in reducing the incidence of preterm PE, SGA and placental dysfunction and preterm birth overall [10,11,12,13]. Moreover, UtA-PI is a major component of other existing predictive models for placenta-mediated adverse pregnancy outcomes, including fetal growth restriction (FGR) and SGA neonates [9,14,15,16,17,18]. Its routine use in clinical practice contributes to more precise risk prediction [19], with the potential to improve maternal-fetal outcomes while optimizing resource allocation [9,10,14,15,16,17].

ART-conceived pregnancies carry a higher risk of placental dysfunction and related adverse outcomes, including a significantly increased incidence of PE and SGA [20,21]. Given the recognized role of UtA-PI in assessing placental perfusion, its application in ART pregnancies warrants scrutiny. Emerging evidence suggests that UtA-PI measurements may differ between ART-conceived and spontaneously conceived (SC) pregnancies, raising concerns about the appropriateness of applying standards derived from SC populations to ART cases [22]. Additionally, most studies focused on UtA-PI levels in ART pregnancies did not consider the different ART techniques. Pooling all ART techniques into the generic category may affect accurate risk assessment. The current body of literature exploring UtA-PI in ART pregnancies is limited and often inconsistent [9,23,24,25]. Given the established link between ART and increased risk for placental dysfunction-related disorders and the pivotal role of UtA-PI as a predictive biomarker for placental function, it is essential to improve our understanding of how UtA-PI varies across different ART modalities.

The aim of this meta-analysis was to systematically synthesize and quantitatively assess differences in first and second trimester UtA-PI measurements between ART pregnancies and SC and stratify the analyses for the different ART modalities. We also aimed to examine in our investigated population how these observed UtA-PI differences in various ART modalities align with their respective risks for outcomes associated with placental dysfunction, such as PE and SGA neonates.

## 2. Materials and Methods

The study was conducted in accordance with the Preferred Reporting Items for Systematic reviews and Meta-Analyses (PRISMA) [26] (Supplementary File S1) and the Meta-analysis Of Observational Studies in Epidemiology (MOOSE) guidelines [27]. The protocol was prospectively registered on the International Platform of Registered Systematic Review and Meta-analysis Protocols (INPLASY). Institutional review board approval or patient consent was not required as this study involved aggregated, previously published data.

### 2.1. Eligibility Criteria

Eligible studies were observational and investigated differences in UtA-PI measurements between SC and ART methods in singleton pregnancies. Studies included UtA-PI Doppler measurements from either the first (until the 13^+6^ gestational week) or second trimester (14^+0^–23^+6^ gestational weeks). The study population comprised women who conceived via ART, while the control population comprised SC pregnancies. Both groups should have UtA Doppler measurements. Studies involving participants under 18 years of age were excluded.

Case reports, small case series, letters to the editor, animal studies, conference proceedings, abstracts and review articles were not included as they lack predefined important information that is necessary for the assessment of study limitations and the quality of evidence is low.

### 2.2. Information Sources and Search Strategy

A comprehensive literature search was conducted to identify studies comparing mean UtA-PI Doppler measurements between SC pregnancies and those achieved through various ART methods and protocols. The search covered MEDLINE (via PubMed), Scopus, and the Cochrane Central Register of Controlled Trials from inception to 25 June 2025. Search strategies were customized for each database to capture studies relevant to uterine artery Doppler assessment in the context of ART. PubMed search string was “((Uterine Artery) OR (UtA)) AND ((((((((Assisted Reproductive Techniques) OR (ART)) OR (Assisted Reproduction)) OR (IVF)) OR (In Vitro Fertilization)) OR (ICSI)) OR (Intracytoplasmic Injection)) OR (Embryo Transfer))”.

Additional sources were identified through Google Scholar, and the reference lists of eligible studies and relevant review articles were manually screened. Only studies published in English were considered for inclusion.

### 2.3. Study Selection

The process of study selection was conducted in three sequential stages. First, the titles and abstracts of all electronic papers were examined to assess their potential eligibility. Secondly, articles that met or were presumed to meet the eligibility criteria were retrieved in full text. Thirdly, we selected the studies that satisfied the eligibility criteria. Study selection was performed by two authors independently, while any potential discrepancies were resolved through discussion or consultation with a third reviewer.

### 2.4. Outcomes

The primary outcome of this review was the standardized mean difference (SMD) in UtA-PI between SC pregnancies and those resulting from ART during the first and second trimesters. Recognizing that UtA-PI measurements are sensitive to gestational age and other confounding factors, we performed relative analyses to take them into account. These analyses utilized gestational age-standardized indices such as Multiples of the Median (MoMs) and effect measures from multivariable models. The aim was to verify if the trends observed in the primary analysis remained after these adjustments.

Interpreting UtA-PI differences between ART and SC pregnancies requires careful consideration. Finding lower UtA-PI values among ART modalities, on its own, is not necessarily clinically relevant, as it could simply indicate better placental perfusion in these pregnancies. However, it is known that ART is often associated with an increased prevalence of adverse perinatal outcomes linked to placental dysfunction [20,21,28]. Therefore, we considered it crucial to meta-analyze key adverse outcomes relevant to placental dysfunction, such as PE and SGA, as well as birthweight, from the same populations in which UtA-PI was measured. This approach allows us to observe how the risk of these pregnancy complications trends among the different ART methods. The hypothesis was that if ART subgroups with lower UtA-PI concurrently exhibit higher rates of adverse outcomes, it would suggest first, that the UtA-PI is influenced by the specific ART methods, protocols, or associated treatments, and second, that other mechanisms beyond placental perfusion and function are involved in the development of PE or SGA.

### 2.5. Data Extraction

Data were extracted using a pre-defined form in Microsoft Excel. Two reviewers independently conducted data extraction, with discrepancies resolved through discussion or consultation with a third reviewer. Extracted data included study characteristics, population characteristics (inclusion/exclusion criteria), Doppler measurement details (timing of Doppler, specific UtA-PI measurement), outcome data (UtA-PI measurements, MoM, adjusted covariates from multivariable linear regressions, and raw data for PE, SGA and birthweight) and ART methods and protocols including whether conception occurred via IVF, ICSI, or ovulation induction/intrauterine insemination (OI/IUI); whether oocytes were autologous or donor-derived; the type of embryo transfer (ET) (fresh or frozen); and the endometrial preparation method (natural or artificial cycle). When studies had overlapping cohorts, data from the most recent and comprehensive publication were utilized.

### 2.6. Quality and Risk of Bias Assessment

The methodological quality of each study was evaluated using the Newcastle-Ottawa Scale (NOS) for observational studies [29]. This assessment focused on three domains: selection of participants, comparability of groups, and ascertainment of outcome, with a maximum of nine stars indicative of higher quality.

Risk of bias within individual studies was assessed using the Quality In Prognosis Studies (QUIPS) tool [30]. Three reviewers independently assessed each study across six domains as outlined by QUIPS [30]. Each domain was categorized as low, moderate, or high risk of bias. Discrepancies in quality assessment and bias rating were resolved through consensus.

### 2.7. Data Synthesis and Statistical Analysis

This was an aggregate data meta-analysis based on study-level summary statistics. In cases where studies reported outcomes at multiple time-points or from overlapping cohorts, we included only one observation per analysis to prevent double counting. However, when studies provided data for distinct, non-overlapping ART subgroups (e.g., fresh versus frozen ET), we included each subgroup separately within the relevant stratified analysis. UtA-PI was analyzed as a continuous variable. When heterogeneous continuous variables were included in an analysis, the results were presented as SMDs, which express effect sizes in units of the pooled standard deviation across studies. When the same type of continuous measurements was analyzed, we presented the results as mean differences (MD). For continuous variables, we employed the inverse variance as our statistical method. For categorical outcomes, our effect measure was risk ratio (RR) and the statistical method used was Mantel–Haenszel. Given the expected variability among studies, a random-effects model (DerSimonian and Laird method) was employed for all meta-analyses. Between-study heterogeneity was assessed using the Cochrane Q test (with *p* < 0.1 indicating significant heterogeneity) and quantified with the I^2^ statistic. Publication bias was assessed visually via funnel plots and statistically using Egger’s test for our primary analyses if more than ten studies were included. In particular, Egger`s test is a linear regression analysis that considers the study-specific effect estimates and their standard errors which are weighted by their inverse variance.

All statistical analyses were conducted using Review Manager (RevMan 5.4.1, The Cochrane Collaboration, 2020.) and R programming language (version 4.4.2).

### 2.8. Confounders and Sensitivity Analysis

We conducted additional analyses to ensure our findings were robust regarding confounding; first, since UtA-PI measurements are sensitive to gestational age, and the exact timing of measurement can influence results, we performed an analysis using gestational age-adjusted indices, specifically MoMs, only for the first trimester for which we had sufficient data.

Second, to account further for potential confounding factors, we extracted regression coefficients from multivariable models in individual studies (Table 1). We then performed inverse-variance weighted meta-analyses using these coefficients (either for UtA-PI or log_10_UtA-PI). Since these multivariable models adjusted for different confounders across studies, the resulting effect estimates are not directly comparable and cannot be used for inference. However, they can still be useful to assess whether the direction and consistency of the association align with the findings from our primary analysis, serving as a supplementary analysis.

Finally, we performed a risk of bias sensitivity analysis, excluding studies that were deemed to have a high risk of bias.

### 2.9. Subgroup Analyses

To thoroughly investigate method-specific vascular profiles through UtA-PI differences across diverse reproductive pathways, we conducted comprehensive subgroup analyses for all investigated outcomes. These analyses involved stratifying pregnancies by specific ART methods and protocols, including cases of OI/IUI, the use of donor or autologous oocytes, fresh or frozen ET, and natural or artificial endometrial preparation. Pregnancies resulting from IVF/ICSI for which further detailed information regarding oocyte origin, specific protocol, or other relevant factors was unavailable were grouped together into a broader, common IVF/ICSI category.

## 3. Results

**Figure 1:**
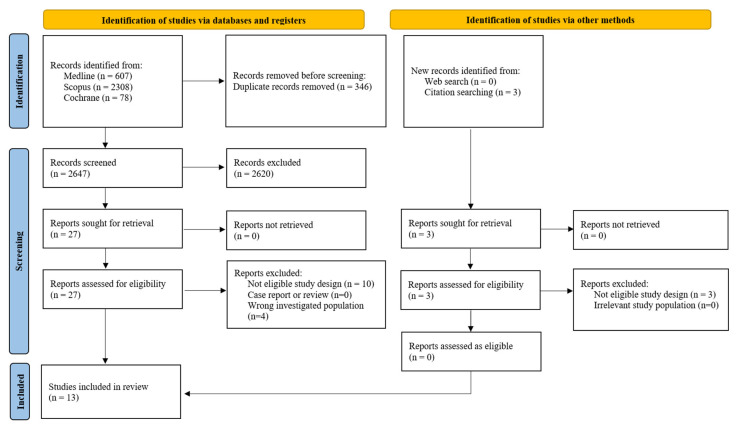
Flowchart of the selection process of the included studies.

A total of 2993 records were identified through the initial database search. After removing 346 duplicates, 2647 unique titles and abstracts were screened, of which 2620 were excluded as not relevant. The remaining 27 studies underwent full-text review to assess eligibility. In parallel, manual reference list screening and targeted supplementary searches identified three additional studies for potential inclusion. Following detailed full-text evaluation, 17 studies were excluded, primarily due to the absence of a spontaneously conceived comparison group or because Doppler measurements were taken before clinical pregnancy was established (Supplementary Table S1). Ultimately, 13 cohort studies met all predefined eligibility criteria and were included in the final meta-analysis. A summary of the study selection process is presented in Figure 1, and the main characteristics of the included studies are outlined in Table 1.

### 3.1. Quality of the Included Studies

The methodological quality of the 13 included studies, as assessed by the Newcastle–Ottawa Scale, was generally robust, though not without limitations. Eight studies (62%) achieved the maximum score of 9 stars, and the remaining five (38%) were awarded 7 stars, indicative of acceptable quality, albeit with notable methodological shortcomings. All studies met the criteria across the Selection and Outcome domains; however, five studies did not adequately address comparability, reflecting insufficient control for confounding variables (Table 2).

### 3.2. UtA PI Differences in the First Trimester

The meta-analysis assessing the SMDs in UtA-PI in the first trimester included 4966 pregnancies conceived via ART. A significant reduction in UtA-PI was observed in the IVF/ICSI oocyte donation (SMD = −0.70; 95% CI: −1.21 to −0.18; I^2^ = 93%) and in frozen ET in artificial cycles subgroup (SMD = −0.69; 95% CI: −1.00 to −0.39; I^2^ = 58%).

Other subgroups showed no statistically significant association with UtA-PI. These included the IVF/ICSI autologous subgroup (SMD = −0.27; 95% CI: −0.91 to 0.38; I^2^ = 94%), the IVF/ICSI group (SMD = −0.20; 95% CI: −0.53 to 0.13; I^2^ = 70%), fresh ET (SMD = 0.29; 95% CI: −0.18 to 0.77; I^2^ = 90%), OI/IUI (SMD = 0.07; 95% CI: −0.03 to 0.17), natural cycle frozen transfers (SMD = −0.07; 95% CI: −0.18 to 0.04), and IVF/ICSI autologous frozen transfers (SMD = 0.03; 95% CI: −0.62 to 0.67).

**Figure 2:**
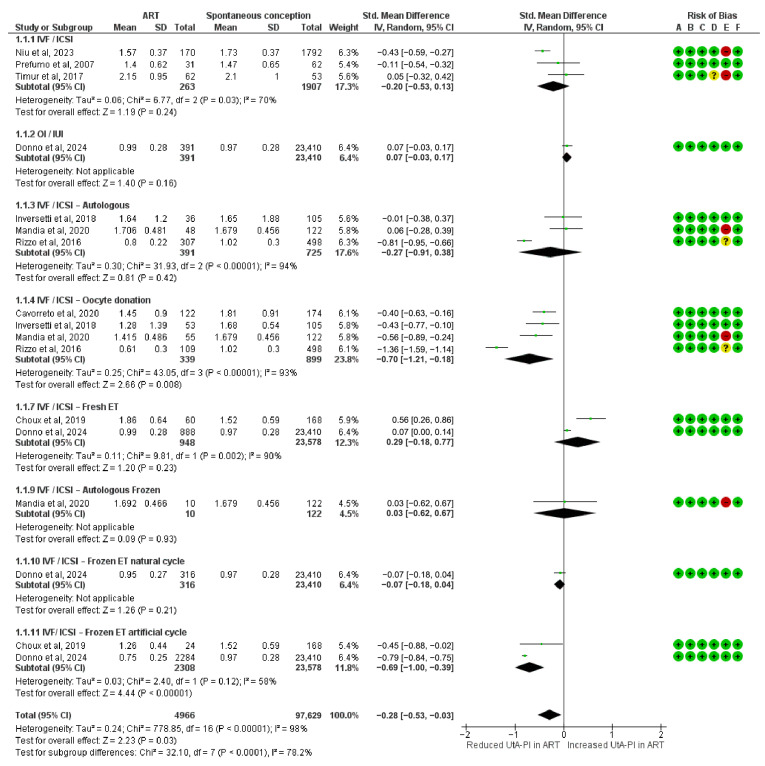
Forest plot analyzing the standardized mean differences in first-trimester uterine artery pulsatility index between ART and spontaneously conceived pregnancies. Abbreviations: ART, assisted reproductive technology; ET, embryo transfer; ICSI, intracytoplasmic sperm injection; IUI, intrauterine insemination; IVF, in vitro fertilization; OI, ovulation induction; SD, standard deviation; Std, standardized; UtA-PI, uterine artery pulsatility index [23,24,25,33,34,37,38,39].

The overall pooled SMD in UtA-PI during the first trimester was −0.28 (95% CI: −0.53 to −0.03), indicating a modest but statistically significant reduction in ART-conceived pregnancies compared to spontaneous conceptions. Subgroup differences were statistically significant (Chi^2^ = 32.10, df = 7, *p* < 0.0001; I^2^ = 78.2%) (Figure 2).

### 3.3. Risk of Bias Sensitivity Analysis

This sensitivity analysis, after excluding the high-risk-of-bias studies, included 4621 pregnancies conceived via ART. A statistically significant reduction in UtA-PI was observed in the IVF/ICSI oocyte donation (SMD = −0.74; 95% CI: −1.41 to −0.06; I^2^ = 95%) and in IVF/ICSI frozen ET in artificial cycles subgroup (SMD = −0.69; 95% CI: −1.00 to −0.39; I^2^ = 58%).

Other subgroups showed no statistically significant association with UtA-PI. These included the IVF/ICSI group (SMD = −0.11; 95% CI: −0.54 to 0.32), IVF/ICSI autologous (SMD = −0.43; 95% CI: −1.21 to 0.36; I^2^ = 93%), IVF/ICSI fresh ET (SMD = 0.29; 95% CI: −0.18 to 0.77; I^2^ = 90%), OI/IUI (SMD = 0.07; 95% CI: −0.03 to 0.17), and IVF/ICSI frozen transfers in natural cycles (SMD = −0.07; 95% CI: −0.18 to 0.04).

**Figure 3:**
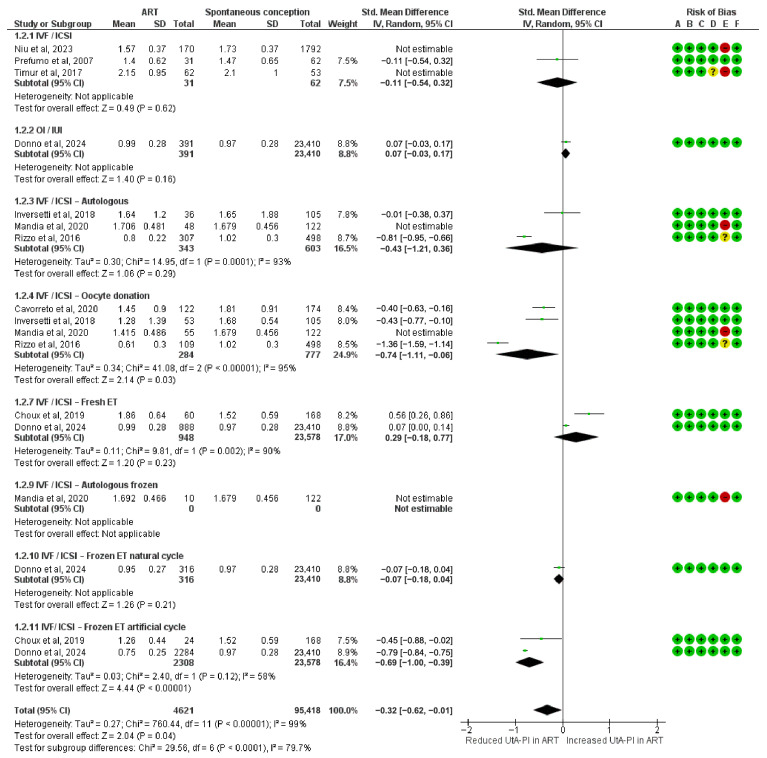
Forest plot analyzing the standardized mean differences in first-trimester uterine artery pulsatility index between ART and spontaneously conceived pregnancies, excluding studies with a high risk of bias. Abbreviations: ART, assisted reproductive technology; ET, embryo transfer; ICSI, intracytoplasmic sperm injection; IUI, intrauterine insemination; IVF, in vitro fertilization; OI, ovulation induction; SD, standard deviation; Std, standardized; UtA-PI, uterine artery pulsatility index [23,24,25,33,34,37,38,39].

The overall pooled SMD in UtA-PI during the first trimester was −0.32 (95% CI: −0.62 to −0.01), indicating a statistically significant reduction in ART-conceived pregnancies when restricting the analysis to studies with low or moderate risk of bias. Subgroup differences remained statistically significant (Chi^2^ = 29.56, df = 6, *p* < 0.0001; I^2^ = 79.7%) (Figure 3). No change was noted from the main analysis.

### 3.4. UtA-PI Differences in Second Trimester

The meta-analysis assessing the SMDs in UtA-PI during the second trimester included 1326 pregnancies conceived via ART. A significant reduction in UtA-PI was observed in the IVF/ICSI with oocyte donation (SMD = −0.46; 95% CI: −0.65 to −0.26; I^2^ = 0%).

Other subgroups showed no statistically significant association with UtA-PI. These included IVF/ICSI (SMD = −0.14; 95% CI: −0.36 to 0.07; I^2^ = 87%) and OI/IUI (SMD = 0.04; 95% CI: −0.18 to 0.26).

**Figure 4:**
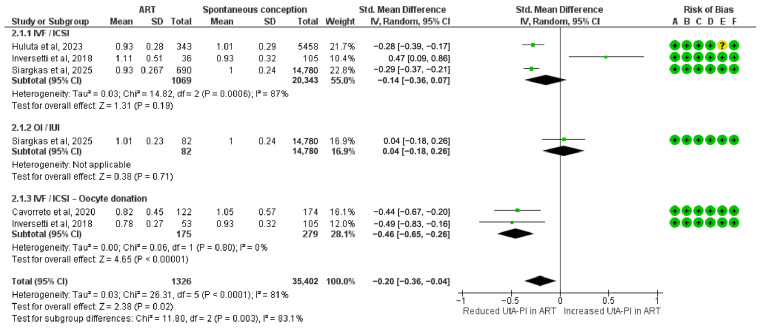
Forest plot analyzing the standardized mean differences in second-trimester uterine artery pulsatility index between ART and spontaneously conceived pregnancies. Abbreviations: ART, assisted reproductive technology; ET, embryo transfer; ICSI, intracytoplasmic sperm injection; IUI, intrauterine insemination; IVF, in vitro fertilization; OI, ovulation induction; SD, standard deviation; Std, standardized; UtA-PI, uterine artery pulsatility index [22,32,36,37].

The overall pooled SMD in UtA-PI during the second trimester was −0.20 (95% CI: −0.36 to −0.04), indicating a modest but statistically significant reduction in ART-conceived pregnancies compared to spontaneous conceptions. Subgroup differences were statistically significant (Chi^2^ = 11.80, df = 2, *p* = 0.003; I^2^ = 83.1%) (Figure 4).

### 3.5. Meta-Analysis of UtA-PI MoM Values

The meta-analysis assessing standardized UtA-PI MoM values across the first trimester included 4295 pregnancies conceived via ART. A significant reduction in UtA-PI MoM values was observed in the IVF/ICSI oocyte donation (MD = −0.41; 95% CI: −0.47 to −0.35), the frozen ET in artificial cycles (MD = −0.22; 95% CI: −0.23 to −0.21), and the IVF/ICSI subgroup (MD = −0.22; 95% CI: −0.28 to −0.16). A small but statistically significant increase was also observed in the IVF/ICSI fresh ET subgroup (MD = 0.02; 95% CI: 0.00 to 0.04).

Other subgroups showed no statistically significant association with UtA-PI. These included OI/IUI (MD = 0.02; 95% CI: −0.01 to 0.05), and IVF/ICSI frozen ET in natural cycles (MD = −0.02; 95% CI: −0.05 to 0.01).

**Figure 5:**
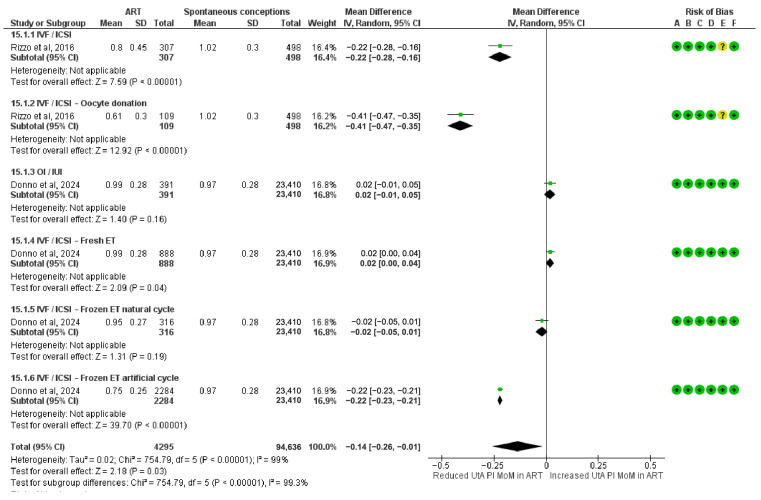
Forest plot analyzing the mean differences in first-trimester standardized uterine artery pulsatility index multiples of the median between ART and spontaneously conceived pregnancies. Abbreviations: ART, assisted reproductive technology; ET, embryo transfer; ICSI, intracytoplasmic sperm injection; IUI, intrauterine insemination; IVF, in vitro fertilization; OI, ovulation induction; SD, standard deviation; UtA-PI, uterine artery pulsatility index [23,34].

The overall pooled MD in standardized UtA-PI in the first trimester was −0.14 (95% CI: −0.26 to −0.01), indicating a modest but statistically significant reduction in ART-conceived pregnancies compared to spontaneous conceptions. Subgroup differences were highly significant (Chi^2^ = 754.79, df = 5, *p* < 0.00001; I^2^ = 99.3%) (Figure 5).

### 3.6. Meta-Analysis of the Adjusted Coefficients from Multivariable Analyses

As a supplementary analysis, we examined trends in UtA-PI differences between ART and SC pregnancies using coefficients derived from multivariable models reported in the included studies (Table 1). These models adjusted for different sets of confounders, and thus the resulting effect estimates are not directly comparable nor suitable for pooled interpretation. However, the analysis provides insight into whether the overall direction and consistency of the associations align with the primary findings.

A trend toward reduced UtA-PI was again observed in the overall ART group and in the IVF/ICSI oocyte donation and IVF/ICSI frozen ET in artificial cycles subgroups. In contrast, minimal or non-significant associations were seen in the remaining subgroups. These trends are consistent with the subgroup-specific patterns reported in the primary unadjusted and MoM-based analyses. The corresponding forest plot is presented in Supplementary Figure S1.

### 3.7. Risk for PE

This meta-analysis assessed the risk of PE and included 6795 pregnancies conceived via ART. A significantly increased risk of PE was observed in the IVF/ICSI oocyte donation (RR = 6.11; 95% CI: 3.35 to 11.17; I^2^ = 0%), the IVF/ICSI frozen ET in artificial cycles (RR = 3.45; 95% CI: 1.51 to 7.90; I^2^ = 14%), the IVF/ICSI (RR = 2.61; 95% CI: 2.01 to 3.39; I^2^ = 4%), and the IVF/ICSI fresh ET subgroup (RR = 1.63; 95% CI: 1.06 to 2.51; I^2^ = 0%).

Other subgroups showed no statistically significant association with PE. These included OI/IUI (RR = 1.35; 95% CI: 0.78 to 2.33; I^2^ = 27%), IVF/ICSI autologous (RR = 1.85; 95% CI: 0.92 to 3.73) and IVF/ICSI frozen ET in natural cycles (RR = 1.15; 95% CI: 0.48 to 2.77).

**Figure 6:**
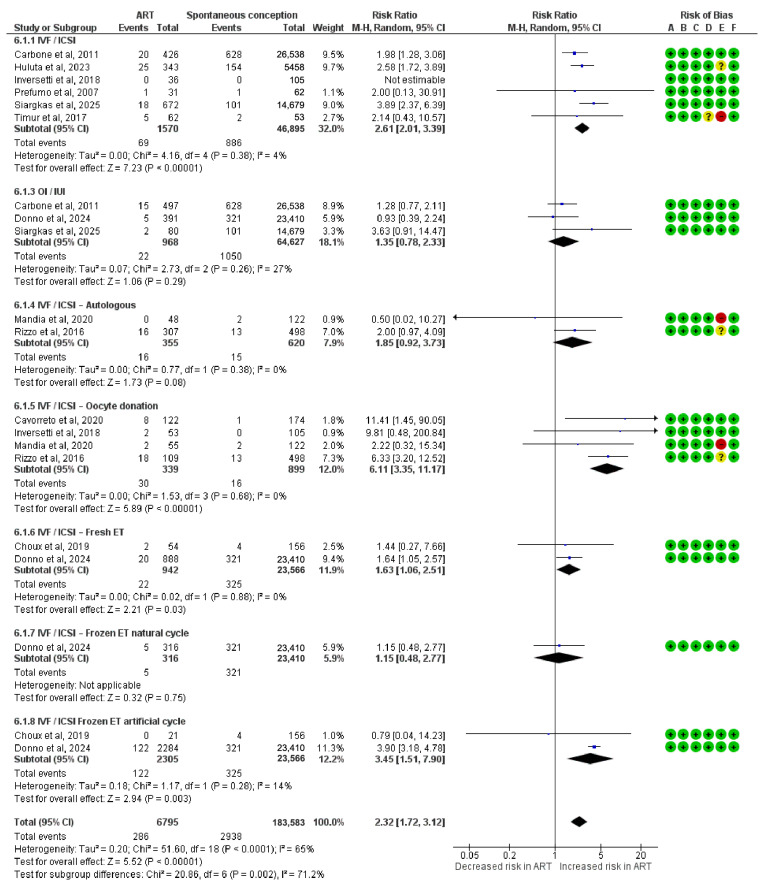
Forest plot analyzing the risk ratios for preeclampsia between ART and spontaneously conceived pregnancies. Abbreviations: ART, assisted reproductive technology; ET, embryo transfer; ICSI, intracytoplasmic sperm injection; IUI, intrauterine insemination; IVF, in vitro fertilization; OI, ovulation induction [22,23,24,31,32,33,34,36,37,38,39].

The overall pooled risk ratio for PE was 2.32 (95% CI: 1.72 to 3.12), indicating a significantly elevated risk in ART-conceived pregnancies. Subgroup differences were statistically significant (Chi^2^ = 20.86, df = 6, *p* = 0.002; I^2^ = 71.2%) (Figure 6).

### 3.8. Risk for SGA Neonates

This meta-analysis assessed the risk of delivering an SGA neonate and included 1358 pregnancies conceived via ART. A significantly increased risk of SGA was observed in the IVF/ICSI fresh ET subgroup (RR = 2.48; 95% CI: 1.12 to 5.50).

Other subgroups showed no statistically significant association with the risk of SGA. These included the IVF/ICSI group (RR = 1.60; 95% CI: 0.81 to 3.15; I^2^ = 86%), OI/IUI (RR = 1.17; 95% CI: 0.68 to 2.02), IVF/ICSI autologous (RR = 1.35; 95% CI: 0.90 to 2.01), IVF/ICSI oocyte donation (RR = 0.98; 95% CI: 0.59 to 1.62; I^2^ = 0%), and IVF/ICSI frozen ET in artificial cycle (RR = 0.72; 95% CI: 0.10 to 5.23).

**Figure 7:**
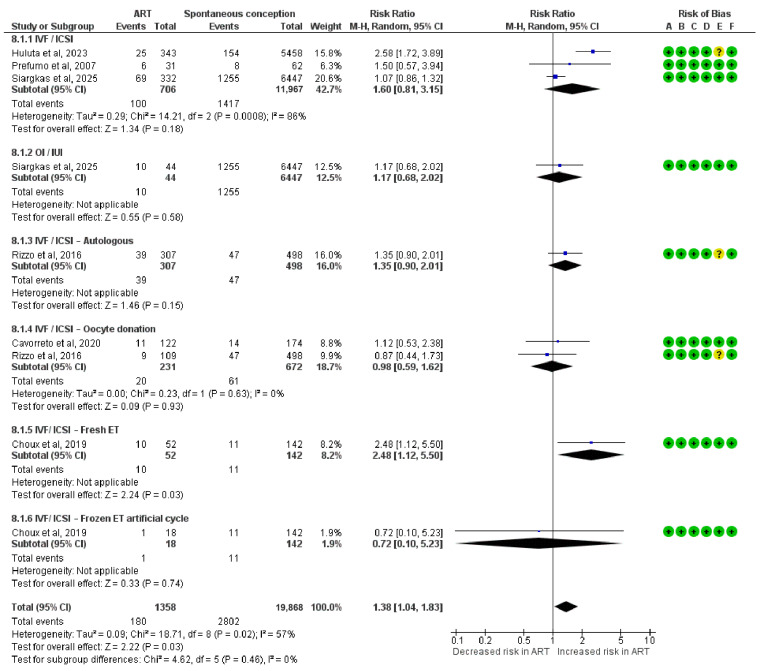
Forest plot analyzing the risk ratios for small for gestational age neonates between ART and spontaneously conceived pregnancies. Abbreviations: ART, assisted reproductive technology; ET, embryo transfer; ICSI, intracytoplasmic sperm injection; IUI, intrauterine insemination; IVF, in vitro fertilization; OI, ovulation induction [22,23,32,33,36,39].

The overall pooled risk ratio for SGA was 1.38 (95% CI: 1.04 to 1.83), indicating a modest but statistically significant increased risk in ART-conceived pregnancies. Subgroup differences were not statistically significant (Chi^2^ = 4.62, df = 5, *p* = 0.46; I^2^ = 0%) (Figure 7).

### 3.9. Mean Difference in Birthweight

This meta-analysis assessed mean differences in birthweight and included 5040 pregnancies conceived via ART. A statistically significant reduction in birthweight was observed in the IVF/ICSI fresh ET subgroup (MD = −148.79 g; 95% CI: −256.57 to −41.02; I^2^ = 48%).

Other subgroups showed no statistically significant association with birthweight. These included IVF/ICSI (MD = −205.61 g; 95% CI: −426.51 to 15.30; I^2^ = 95%), OI/IUI (MD = −86.39 g; 95% CI: −185.70 to 12.92; I^2^ = 59%), IVF/ICSI oocyte donation (MD = 40.00 g; 95% CI: −88.50 to 168.50), IVF/ICSI frozen ET in natural cycles (MD = −1.00 g; 95% CI: −52.05 to 50.05), and IVF/ICSI frozen ET in artificial cycle (MD = 90.02 g; 95% CI: −155.22 to 335.26).

**Figure 8:**
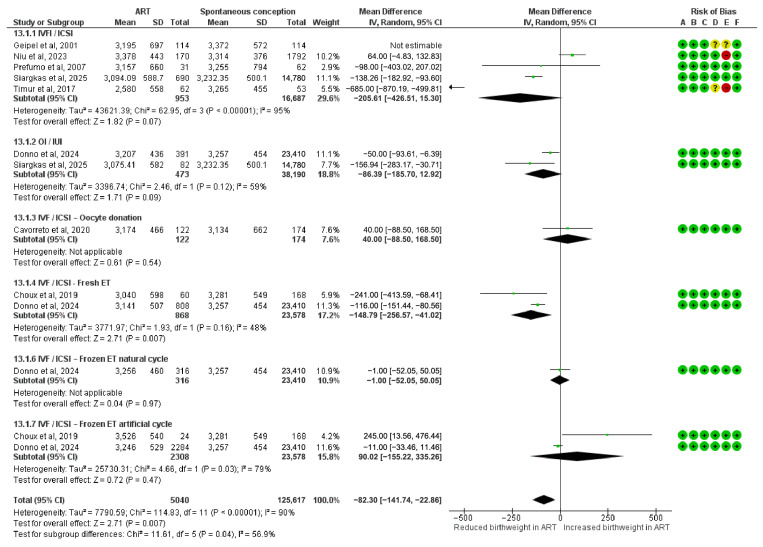
Forest plot analyzing the mean differences in birthweight between ART and spontaneously conceived pregnancies. Abbreviations: ART, assisted reproductive technology; ET, embryo transfer; ICSI, intracytoplasmic sperm injection; IUI, intrauterine insemination; IVF, in vitro fertilization; OI, ovulation induction [22,24,25,32,33,34,39,40].

The overall pooled mean difference in birthweight was −82.30 g (95% CI: −141.74 to −22.86), indicating a modest but statistically significant reduction in ART-conceived pregnancies. Subgroup differences were statistically significant (Chi^2^ = 11.61, df = 5, *p* = 0.04; I^2^ = 56.9%) (Figure 8).

### 3.10. Publication Bias

**Figure 9:**
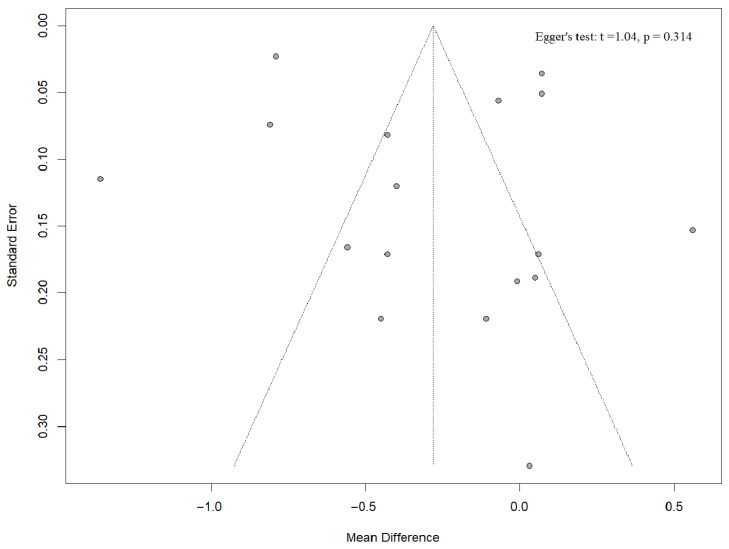
Funnel plot and Egger’s test for publication bias in the first-trimester UtA-PI meta-analysis [23,24,25,33,34,37,38,39].

For the first-trimester UtA-PI meta-analysis, the funnel plot is visually symmetrical and Egger’s regression intercept is non-significant (*p* = 0.314), so there is no detectable small-study or publication bias. Although some studies are outside the margins, they are symmetrical on both sides, not depicting any possible publication bias (Figure 9). We did not run the same tests for the second-trimester analysis because it includes fewer than ten studies, below the threshold at which funnel-plot and Egger statistics have enough power to be meaningful.

## 4. Discussion

### 4.1. Primary Findings

Overall, ART pregnancies showed a reduction in UtA-PI both in the first and the second trimester of pregnancy. This difference was driven primarily by two subgroups, IVF/ICSI with oocyte donation and frozen ET with artificial cycle. These findings persisted when we analyzed UtA-PI MoMs and coefficients from multivariable models. Interestingly, the subgroups with the lowest UtA-PI, oocyte donation and frozen ET with artificial cycle bore the highest burden of PE, while the overall ART pooled risk for PE was also elevated. The IVF/ICSI subgroup showed no significant difference in raw UtA-PI values, compared to SC. However, IVF/ICSI had significantly lower UtA-PI MoM values, yet this group also exhibited a more than two-and-a-half-fold excess risk of PE.

### 4.2. Interpretation

To our knowledge, this is the first meta-analysis to examine the relationship between ART and UtA-PI in a trimester-specific and method-specific manner. Our findings indicate that ART pregnancies generally exhibit lower UtA-PI values in both the first and second trimesters. This observation was further corroborated by our risk of bias sensitivity analysis, which revealed an even more pronounced negative SMD. This trend persisted after adjusting for other confounders in multivariable models and when UtA-PI was standardized as MoMs. Typically, lower UtA-PI values are considered a positive prognostic indicator for pregnancy outcomes. Elevated UtA-PI in the first trimester is associated with an increased risk of PE and FGR [41], while elevated UtA-PI in the second trimester is linked to an increased risk of PE, SGA neonates, cesarean section, and admission to neonatal intensive care unit [42]. However, in our investigated population, we found an increased risk for both PE and SGA neonates among the ART pregnancies, despite the lower UtA-PI we noted. Additionally, we noted lower birthweight in the ART group. However, the considerable heterogeneity within this broad ART group suggests that a discussion of these overall associations is less informative than a detailed examination of associations within specific subgroups.

### 4.3. IVF/ICSI Subgroup

IVF/ICSI with no further definition of protocol or method depicted no statistically significant reduction in nominal terms when compared to SC in the first and second trimester. However, the MoM-standardized index depicted a statistically significant reduction in MD, while the coefficients of the adjusted multivariable models exhibited no significant difference. These findings are important as they show that the broad IVF/ICSI group has similar UtA-PI measurements to SC and may help reconcile conflicting results reported in the literature, particularly in the second trimester. For instance, the study by Inversetti et al. reported a statistically significant increase in UtA-PI among IVF/ICSI pregnancies compared to SC in the second trimester [37], while two other studies indicated a reduction [22,36]. More critically, despite having comparable first and second trimester UtA-PI values compared to the SC, the IVF/ICSI group exhibited a markedly higher risk for PE, with an additional, though non-significant, elevation in the risk for SGA neonates.

This suggests a complex and potentially counterintuitive relationship; even in the presence of lower UtA Doppler indices, typically associated with improved uteroplacental perfusion, IVF/ICSI pregnancies may remain at elevated risk for placental dysfunction-related complications [43,44]. Currently, there is no definitive theory to explain this phenomenon. We hypothesize that either the lower UtA-PI in IVF/ICSI pregnancies is influenced by specific ART methods, protocols, or associated treatments, or that other mechanisms contribute to the development of PE.

### 4.4. Oocyte Donation and Autologous Transfer Subgroups

Our subgroup analyses further revealed that the biological origin of the embryo plays a critical role in shaping UtA-PI profiles. IVF/ICSI with oocyte donation consistently exhibited the most pronounced reductions in UtA-PI relative to SC, with a first-trimester SMD of −0.70 and a second-trimester SMD of −0.46. These differences were also confirmed in the MoM standardized index in the first trimester (MD = −0.41) and the trend also persisted in the analysis of adjusted coefficients from multivariable regressions. These significantly lower UtA-PI measurements are generally associated with better placental perfusion and decreased risk for adverse outcomes like PE [45]. However, this subgroup exhibited the highest risk for PE (RR = 6.1).

This discrepancy between expected and observed PE risk in oocyte donation pregnancies highlights a critical clinical concern. The significant MoM reduction in the oocyte donation group of approximately 0.4 units means that their measured UtA-PI MoM values are shifted downwards compared to SC. To put this in context, based on the study of Tan et al., pregnant women with a median UtA-PI MoM of 1.4 are at increased risk for developing PE around 30–32 weeks [45]. Given our results, women after successful oocyte donation tend to have lower UtA-PI MoM of about 0.4 units compared to SC and might therefore present with values closer to the population median of 1.0 despite their elevated risk for PE. Taking into account that at the moment, the models for PE risk assessment do not stratify for oocyte donation but for the broader IVF group [45]. This may result in false negative cases in the oocyte donation pregnancies, leading to a lack of appropriate prophylactic treatment, which in turn, may contribute to the alarmingly elevated PE risk we observe in this population.

The discordance between lower UtA-PI and higher PE risk in the oocyte donation group may reflect a fundamental shift in the underlying pathophysiology. The reduced UtA-PI and the improved placental perfusion, in these cases, may reflect pharmacologically mediated vasodilation rather than improved placental function. Specifically, exogenous estrogen administered during endometrial preparation in oocyte donation may induce supraphysiological estrogen levels, leading to vasodilation and reduced uterine vascular resistance [46]. This hormonal environment may also dysregulate extravillous trophoblast invasion and spiral artery remodeling [47]. More specifically, studies conducted in animal models have demonstrated that exogenously elevating circulating estrogen levels during the first trimester can lead to increased concentrations of vascular endothelial growth factor [48]. Such elevated levels might compromise trophoblastic vascular remodeling, thereby exerting a quantifiable detrimental effect on uterine and umbilical hemodynamics, as evidenced by Doppler analysis [49]. In terms of PE risk, an explanation lies in immunological maladaptation. The introduction of donor-derived antigens through oocyte donation may provoke an atypical maternal immune response, compounded by hormonal alterations in IVF cycles [50]. The inability of the maternal cardiovascular system to accommodate the hemodynamic burden of pregnancy may represent an alternative pathogenic pathway [51]. Thus, PE in oocyte donation pregnancies may be more closely tied to maternal systemic dysfunction than to classic placental insufficiency. This hypothesis is further supported by the disparity between the severely elevated risk for PE and no increased risk for SGA in the oocyte donation subgroup which may suggest a good placental function but inability of the maternal cardiovascular system to support the pregnancy [51].

Conversely, autologous IVF/ICSI was not associated with lower UtA-PI and showed non-significant associations with PE and SGA. These findings underscore the substantial heterogeneity within ART methods and suggest that the oocyte or embryo origin substantially influences both Doppler indices and the obstetric outcomes we investigated.

### 4.5. Artificial and Natural Cycle Subgroups

Our findings further support the hypothesis of a hyperestrogenic effect through analysis of ET protocols. In IVF/ICSI pregnancies utilizing artificial endometrial preparation, UtA-PI was significantly reduced in the first trimester compared to SC (SMD = −0.69). Although second-trimester data were unavailable, this reduction was confirmed by the MoM standardized index and the covariates from the multivariable models. Importantly, this group also exhibited increased risks for PE (RR = 3.45). Similarly to the patterns seen with oocyte donation, though to a lesser extent, these disparities may hinder the early diagnosis of high-risk PE pregnancies and the timely administration of prophylactic therapy.

Conversely, IVF/ICSI pregnancies using natural cycles show neither a reduction in UtA-PI nor were they significantly associated with adverse outcomes. Making the hypothesis that reductions in UtA-PI observed in artificial cycle and oocyte donation pregnancies reflect pharmacologically mediated vasodilation rather than improved placental function, more possible [46]. Otherwise, the above associations may imply that PE in these pregnancies is not primarily due to placental dysfunction but rather an insufficiency of the maternal cardiovascular system to support the pregnancy [51]. Future studies are needed to clarify the pathophysiological mechanisms underlying these associations and to determine whether UtA-PI retains predictive utility in hormonally manipulated ART contexts.

### 4.6. Rest of the Subgroups

No significant association was observed between OI/IUI and the UtA-PI. Furthermore, this subgroup, similar to SC, showed no association with PE or SGA.

We did not find a general trend of reduced or increased UtA-PI based on embryo freezing status (fresh or frozen), with the exception of protocol-specific subgroups. In the context of frozen ET, only those utilizing an artificial cycle protocol exhibited a reduction in UtA-PI, whereas those in a natural cycle showed no significant change in UtA-PI compared to SC. Within the frozen ET subgroups, only the artificial cycle protocol group demonstrated an increased risk for PE (RR = 3.45), while the natural cycle group did not. Conversely, pregnancies resulting from fresh ET did not show a significantly different UtA-PI compared to SC. However, fresh ET was associated with a significantly increased risk of PE (RR = 1.63), SGA (RR = 2.48), and lower birthweight (MD = −148.79 g).

These findings highlight distinct patterns regarding the impact of embryo status on perinatal outcomes; both frozen ET in a natural cycle and fresh ET showed no difference in UtA-PI, yet fresh ET was associated with an increased risk for all investigated adverse outcomes, in contrast to frozen ET with a natural cycle. Our findings agree with the relevant literature [52,53] but they warrant further research in order for possible pathophysiologic mechanisms to be identified.

### 4.7. Clinical Implications

These findings might have important clinical implications for ART pregnancy screening and management. There is a notable observation that certain ART subgroups, specifically those involving oocyte donation and frozen ET with an artificial cycle, appear to exhibit lower UtA-PI values while concurrently facing a higher risk of PE. The observed increased risk of PE in various ART subgroups might suggest the need for heightened surveillance and tailored antenatal care strategies for these pregnancies. In particular, counseling for patients undergoing these specific ART procedures could include information regarding the background risk for placental dysfunction and the distinct relationship between uterine artery Doppler indices and obstetric outcomes. Particularly, for oocyte donation and to a lesser extent for artificial cycle frozen ET, we laid out examples of how we may miss pregnancies in these subgroups that are at risk of PE. Clinicians might consider caution and not necessarily interpret lower UtA-PI values as indicative of better placental function or lower risk in these specific ART populations. The current practice of utilizing UtA-PI in first-trimester screening algorithms for PE necessitates UtA-PI adjustment and further stratification for pregnancies conceived via oocyte donation or frozen ET. This is crucial to potentially avoid false negatives among these groups, which are at a particularly higher risk for PE compared to the broader IVF population. Currently, ISUOG guidelines advocate for the use of UtA-PI in first-trimester screening for the prediction of PE; however, the associated predictive models, while adjusting for ART as a general category, fail to incorporate specific adjustments for clinically significant modalities such as oocyte donation and frozen ET [54]. Additionally, several large studies form the FMF group suggest incorporating UtA-PI in the second-trimester prediction of PE and SGA, adjusting for IVF pregnancies in general without any corrections for the different ART types [9,14,15,16,18].

### 4.8. Future Directions

Future research should explore UtA-PI in pregnancies affected by PE versus those unaffected, across different ART modalities, and particularly oocyte donation and frozen ET with artificial cycle groups. Such studies would help determine if ART pregnancies affected by PE inherently have lower UtA-PI compared to the SC, or if the mean UtA-PI for the entire ART group is skewed downwards by the healthy pregnancies. Additionally, dose–response studies investigating the relationship between estrogen levels, UtA-PI, and adverse outcomes among ART groups would provide valuable insights.

### 4.9. Strengths and Limitations

This meta-analysis possesses several strengths. It is, to our knowledge, the first to systematically synthesize differences in UtA-PI between ART and spontaneously conceived pregnancies in a trimester-specific and ART method-specific manner. The study adhered to PRISMA guidelines and included prospective protocol registration, enhancing transparency and methodological rigor. A comprehensive literature search was conducted across multiple databases. The inclusion of various UtA-PI metrics, including raw values, MoMs, and adjusted coefficients from multivariable models, allowed for a robust assessment of UtA-PI differences. Critically, the meta-analysis also contextualized UtA-PI findings by examining key placenta-mediated adverse outcomes (PE and SGA) within the same study populations. Methodological quality and risk of bias were rigorously assessed using established tools like the Newcastle–Ottawa Scale and QUIPS tool.

Despite these strengths, certain limitations should be acknowledged. A primary limitation is the significant between-study heterogeneity observed in many analyses. This variability largely stems from the noticeable non-uniformity in the definition and reporting of ART subgroups across the included studies, as well as the varying time windows of the UtA-PI measurements. The observational nature of the included studies means that residual confounding cannot be entirely excluded, even though relevant analyses using adjusted estimates were performed. A significant confounder that is hard to evaluate is medications more commonly used in ART pregnancies, such as aspirin, low molecular weight heparin, and progesterone, that could affect both UtA-PI and adverse outcomes [46,47,55]. Unfortunately, we were unable to perform a meta-regression to further explore heterogeneity due to an insufficient number of studies and inconsistent data reporting. We hope to conduct this analysis in the future if more high-quality studies with standardized reporting become available. Some subgroup analyses, particularly for specific ART modalities or second-trimester outcomes, were based on a limited number of studies, which can affect the precision and generalizability of these specific findings. Crucially, this study could not establish a direct clinical association between UtA-PI values in various ART modalities and adverse outcomes, as it primarily investigated mean values within these populations due to lack of data on the direct associations between UtA-PI and adverse perinatal outcomes such as PE.

## 5. Conclusions

This meta-analysis reveals that ART singleton pregnancies and most notably those involving oocyte donation and artificial cycle frozen ET, exhibit lower UtA-PI values compared to SC. These findings of lower UtA-PI coexist with significantly elevated risks for PE within these ART subgroups. The interpretation is that either reduced UtA-PI does not reflect improved placental function but is influenced by specific ART treatment, or that placental function is indeed better in ART pregnancies, and other primarily maternal mechanisms contribute to the development of PE. In the clinical front, our results point to a concerning direction; pregnancies from oocyte donation and those utilizing artificial cycle protocols are at very high risk of PE, but we may miss these cases if we overlook the ART-specific technique. Multivariate algorithms, which are now in place for the early identification of PE should be adapted to include ART methods as a risk factor. Also, UtA-PI should be adjusted for ART method so that placental dysfunction reflected in impedance to flow in the uterine arteries is not masked. Further research is imperative to elucidate the underlying pathophysiological mechanisms and to investigate ART-method-specific UtA-PI assessment strategies to improve clinical management and maternal-fetal outcomes.

## Figures and Tables

**Table 1:** Characteristics of the included studies.

Study, Year	Study Period	Study Type	Country	Inclusion Criteria	Exclusion Criteria	ART Methods	UtA Doppler Measurements	Time of the Measurements	Adjustment
Carbone et al., 2011 [31]	March 2006–October 2009	Prospective cohort study	UK	Singleton pregnancies, attending for their routine first hospital visit during pregnancy at King’s College Hospital, London, UK and Medway Maritime Hospital, Kent, UK.	Pregnancies conceived by IUI because data on OI drugs were unavailable, fetal aneuploidies or major defects diagnosed either prenatally or in the neonatal period, and pregnancies ending in termination for psychosocial reasons.	OI, IVF/ICSI	MoM UtA-PI	11^+0^ to 13^+6^ weeks of gestation	Fetal crown-rump length, maternal age, maternal weight, maternal height, racial origin, cigarette smoking, method of conception, history of chronic hypertension, history of diabetes mellitus, parity, family history of preeclampsia
Cavoretto et al., 2020 [32]	January 2013–December 2018	Prospective cohort study	Italy	Singleton pregnancies following IVF/ICSI with OD, and matched naturally conceived controls; light smoking (<5 cigarettes/day) and BMI as matching criteria.	Other ARTs (e.g., autologous IVF/ICSI, IUI, gamete/zygote intra-fallopian transfers), heavy smoking (≥5 cigarettes/day), twin pregnancies, fetal aneuploidies, major fetal defects, spontaneous abortions, insulin-dependent diabetes mellitus (type 1 or 2), cardiac, renal or liver diseases, chronic infections, overt autoimmune diseases (e.g., antiphospholipid syndrome, lupus), chronic hypertension (requiring treatment before pregnancy), chromosomal/genetic abnormalities, cancer history, multiple myomectomy with potential uterine artery damage.	IVF/ICSI with OD	Mean UtA-PI	11^+0^ to 13^+6^, 19^+0^–21^+6^, 26^+0^–33^+6^ gestational weeks	GA, maternal weight, parity
Choux et al., 2019 [33]	1 October 2013–31 January 2015	Retrospective case–control study	France	Singleton IVF pregnancies (fresh ET or frozen ET), with dates between 1 October 2013 and 31 January 2015, followed at Dijon University Hospital for first-trimester ultrasound and placental acquisition.	Pre-existing maternal diseases (diabetes, chronic hypertension), OD, fetal malformation or abnormal karyotype.	IVF with either fresh ET or frozen ET	Mean UtA-PI, Presence of diastolic notching	11^+0^ to 13^+6^ gestational weeks	GA
Donno et al., 2024 [34]	January 2010–May 2023	Retrospective cohort study	Spain	Singleton pregnancies (natural conception or ART) with full pregnancy follow-up from first-trimester screening to delivery.	-	OI/IUI, IVF and fresh ET or frozen ET in natural cycle or frozen ET in artificial cycle	MoM UtA-PI	11^+0^ to 13^+6^ gestational weeks	No
Duijn et al., 2021 [35]	January 2017–March 2018	Prospective cohort study	The Netherlands	Women 18 years and older, before 10 weeks of gestation with a viable singleton pregnancy, were eligible for participation.	Pregnancies after OD, pregnancies resulting in a miscarriage and drop-outs were excluded.	fresh or frozen ET	Mean UtA-PI, RI	Transvaginal at 7th, 9th, 11^+0^ to 13^+6^ gestational weeks; Transabdominal at 22nd week of gestation	GA, parity, periconceptional folic acid supplement use, smoking
Huluta et al., 2023 [36]	August 2019–December 2021	Prospective cohort study	UK	Women with singleton pregnancies attending routine ultrasound at 19+0 to 23+6 weeks, delivering a liveborn fetus without major congenital abnormalities.	Pregnancies with aneuploidy or major fetal abnormality.	IVF/ICSI	MoM UtA-PI	19^+0^ to 23^+6^ gestational weeks	No
Inversetti et al., 2018 [37]	2010–2015	Prospective cohort study	Italy	Consecutive pregnant women (2010–2015) at two tertiary care centers, with different modes of conception.	Non-singleton pregnancy, aneuploidies, history of preeclampsia, history of hypertension or diabetes, autoimmune disease.	autologous ICSI from fresh transfer and heterologous by ICSI-OD	Mean UtA-PI	First trimester at 11^+0^ to 13^+6^ gestational weeks, second trimester at 14^+0^ to 23^+6^ gestational weeks	maternal age, BMI, race, parity, smoking status and GA
Mandia et al., 2020 [38]	January 2014–January 2016	Prospective case–control study	Italy	Singleton pregnancies from nulliparous women with different methods of assisted conception or normal pregnancies, attending for routine first-trimester prenatal risk assessment.	Pre-existing maternal diseases (diabetes, chronic hypertension, renal disease, other significant diseases), fetal structural or chromosomal anomalies, vaginal bleeding or threatened miscarriage, multiple pregnancies or pregnancies with vanishing twins above 6 weeks of development (crown rump length > 10 mm), smoking status, data from patients lost to follow-up.	IVF with OD or autologous oocytes from fresh cycles or autologous oocytes from frozen cycles	Mean UtA-PI	11^+0^ to 13^+6^ gestational weeks	No
Niu et al., 2023 [25]	December 2017–December 2020	Prospective cohort study	China	Healthy women with ongoing first-trimester pregnancy, no tobacco, recreational drug use, or excessive alcohol (>50 mL/week), and adequate nutrition; viable singleton with crown rump length 45–84 mm; normal fetal growth (10th–90th percentile) on growth curves; spontaneous or IVF pregnancies.	Participants with pre-existing maternal diseases (e.g., diabetes, immune, cardiovascular, renal, liver, malignancy, gynecological or endocrine disorders, chronic hypertension, or history of preeclampsia/preterm birth), uterine malformations or surgeries (e.g., myomectomy), adverse pregnancy outcomes (fetal growth restriction, preeclampsia, eclampsia), and fetuses with chromosomal abnormalities, major defects, or spontaneous abortions.	IVF/ICSI	Mean UtA-PI, PSV, End-Diastolic Velocity	11^+0^–13^+6^ gestational weeks	No
Prefumo et al., 2007 [39]	December 2001–April 2005	Prospective case–control study	UK	Women with singleton pregnancies attending routine first-trimester Doppler.	Women with a known medical condition (e.g., diabetes mellitus, connective tissue disease, essential hypertension) or a history of recurrent miscarriage.	IVF/ICSI	Mean RI, PI, presence of notching	11^+0^–13^+6^ gestational weeks	No
Rizzo et al., 2016 [23]	January 2007–January 2014	Prospective cohort study	Italy	Singleton pregnancies with successful recordings of UtA Doppler waveforms, no pre-existing maternal diseases (diabetes, chronic hypertension, renal disease), no fetal anomalies, and exhaustive follow-up (delivered at the unit).	-	IVF with autologous and donor oocytes	MoM UtA-PI	11^+0^–13^+6^ gestational weeks	No
Siargkas et al., 2025 [22]	February 2015–August 2024	Retrospective cohort study	Greece	Consecutive singleton pregnancies receiving antenatal care at our institution, confirmed viable from 20^+0^ weeks until 23^+6^ weeks of gestation, that had also attended for the first trimester nuchal scan in the same department.	Multiple gestations, singleton pregnancies with known fetal genetic or structural anomalies, termination of pregnancy, miscarriages before 23+6 weeks, and incomplete follow-up data.	IVF/ICSI, OI/IUI	Mean UtA-PI	20^+0^–23^+6^ gestational weeks	GA, maternal weight, height, age, parity, smoking status, history of previous cesarean section, pre-existing diabetes mellitus (type I or II), and pre-existing thyroid disease
Timur et al., 2017 [24]	May 2013–June 2015	Prospective case–control study	Turkey	Healthy nulliparous women with singleton pregnancies following IVF, and matched controls with spontaneous singleton pregnancies.	Women with pre-existing diseases (diabetes, chronic hypertension, renal disease), or fetuses with structural or chromosomal anomalies.	IVF/ICSI	Left and right UtA PI, RI	First trimester	No

Abbreviations: ART, Assisted Reproductive Technology; BMI, Body Mass Index; ET, Embryo Transfer; GA, gestational age; ICSI, Intracytoplasmic Sperm Injection; IUI, Intrauterine Insemination; IVF, In Vitro Fertilization; MoM, Multiples of the Median; OD, Oocyte Donation; OI, Ovulation Induction; PI, Pulsatility Index; PSV, Peak Systolic Velocity; RI, Resistance Index; UtA, Uterine Artery.

**Table 2:** Quality of the included studies.

First Author, Year	Study Type	S1	S2	S3	S4	C	O1	O2	O3	Total
Carbone et al., 2011 [31]	Prospective cohort study	b *	a *	a *	a *	a,b **	b *	a *	a *	9
Cavoretto et al., 2020 [32]	Prospective cohort study	b *	a *	a *	a *	a,b **	b *	a *	a *	9
Choux et al., 2019 [33]	Retrospective case–control study	a *	a *	a *	a *	a,b **	a *	a *	a *	9
Donno et al., 2024 [34]	Retrospective cohort study	b *	a *	a *	a *	a,b **	b *	a *	a *	9
Duijn et al., 2021 [35]	Prospective cohort study	a *	a *	a *	a *	a,b **	a *	a *	a *	9
Huluta et al., 2023 [36]	Prospective cohort study	b *	a *	a *	a *	a *	b *	a *	a *	7
Inversetti et al., 2018 [37]	Prospective cohort study	b *	a *	a *	a *	a,b **	b *	a *	a *	9
Mandia et al., 2020 [38]	Prospective case–control study	b *	a *	a *	a *	-	b *	a *	a *	7
Niu et al., 2023 [25]	Prospective cohort study	b *	a *	a *	a *	-	b *	a *	a *	7
Prefumo et al., 2007 [39]	Prospective case–control study	a *	a *	a *	a *	a,b **	a *	a *	a *	9
Rizzo et al., 2016 [23]	Prospective cohort study	b *	a *	a *	a *	a *	b *	a *	a *	7
Siargkas et al., 2025 [22]	Retrospective cohort study	a *	a *	a *	a *	a,b **	a *	a *	a *	9
Timur et al., 2017 [24]	Prospective case–control study	b *	a *	a *	a *	-	b *	a *	a *	7

Abbreviations: a, first answer according to Newcastle–Ottawa Scale; b, second answer according to Newcastle-Ottawa Scale; S, selection; C, comparability; O, outcome; *, attribution of a star according to Newcastle-Ottawa Scale; ** attribution of two stars according to Newcastle-Ottawa Scale.

## Data Availability

Not applicable.

## Supplementary Materials

The following supporting information can be downloaded at: https://www.mdpi.com/article/10.3390/diagnostics15172192/s1. Supplementary Figure S1: Forest plot analyzing the multivariable linear regression coefficients (β) of uterine artery pulsatility index in ART versus spontaneously conceived pregnancies from multivariable analyses.; Supplementary Table S1: Excluded studies that may seem to fulfill our criteria [40,56,57,58,59,60,61,62,63,64,65,66,67,68,69,70,71]; Supplementary File S1: PRISMA_2020_checklist.

## Abbreviations

ART	Assisted Reproductive Technology
CI	Confidence Interval
Df	Degrees of Freedom
ET	Embryo Transfer
ICSI	Intracytoplasmic Sperm Injection
IUI	Intrauterine Insemination
IVF	In Vitro Fertilization
MD	Mean Differences
MoM	Multiples of the Median
OI	Ovulation Induction
PE	Preeclampsia
PI	Pulsatility Index
QUIPS	Quality In Prognosis Studies
RR	Risk Ratio
SC	Spontaneous Conceptions
SD	Standard Deviation
SGA	Small-for-gestational-age
SMD	Standardized Mean Difference
Std	Standardized
UtA	Uterine Artery
UtA-PI	Uterine Artery Pulsatility Index
