# Supplementary material for: Uterine Artery Pulsatility Index in Singleton Pregnancies Conceived via Assisted Reproductive Technology Versus Spontaneous Conception: A Systematic Review and Meta-Analysis
Source: Diagnostics (Basel). 2025 Aug 29;15(17):2192. doi: 10.3390/diagnostics15172192 (PMC12427646; doi:10.3390/diagnostics15172192)
Supplement: Supplementary file 1 [file diagnostics-15-02192-s001.zip › Supplementary figures.pdf]

Supplementary figure S1: Forest plot analyzing the multivariable linear regression coefficients (β) of uterine artery pulsatility index in ART versus spontaneously conceived pregnancies from multivariable analyses.

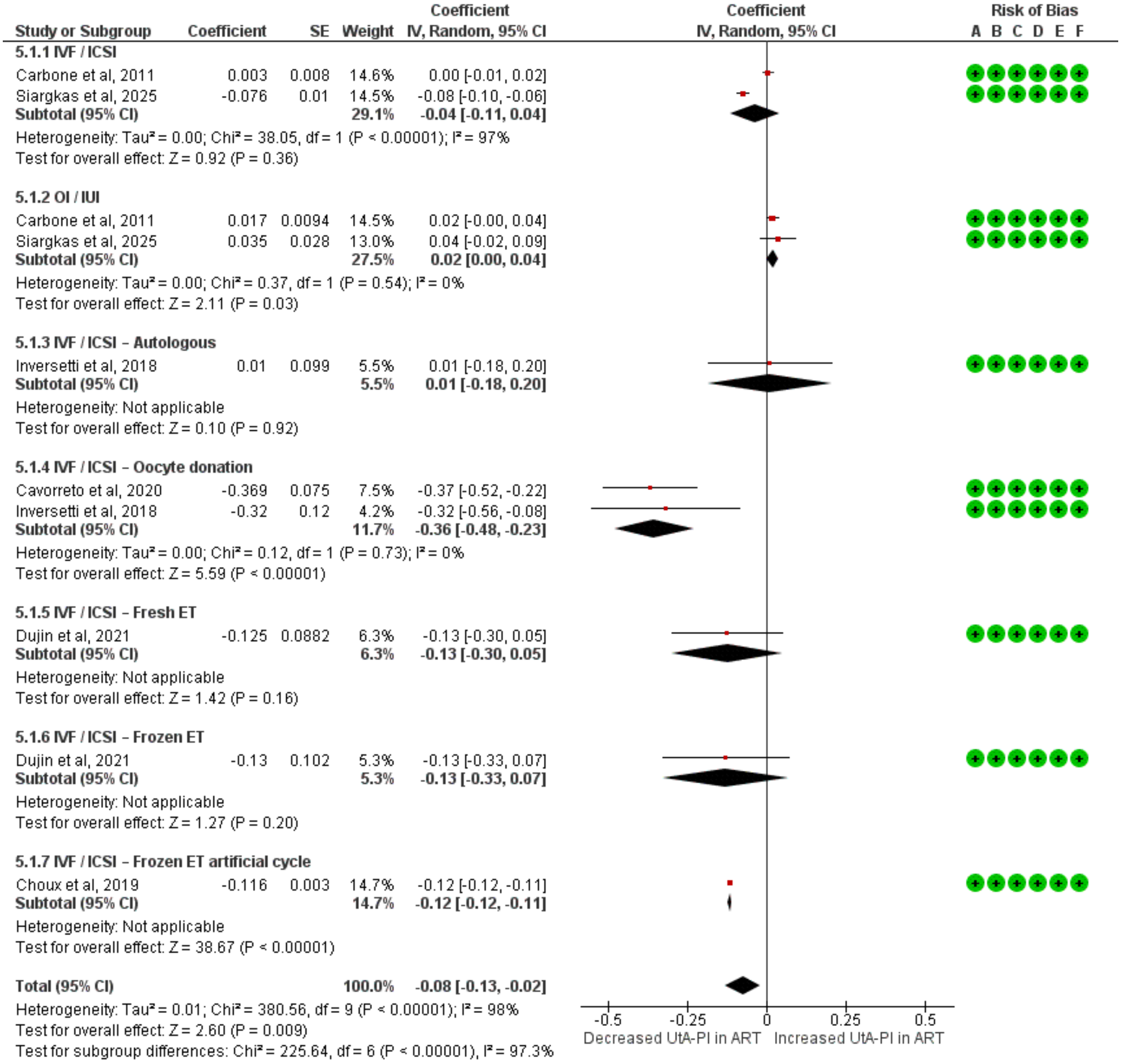

Abbreviations: ART, assisted reproductive technology; ET, embryo transfer; ICSI, intracytoplasmic sperm injection; IUI, intrauterine insemination; IVF, in vitro fertilization; OI, ovulation induction; SE, standard error; UtA-PI, uterine artery pulsatility index
