# Supplementary material for: Uterine Artery Pulsatility Index in Singleton Pregnancies Conceived via Assisted Reproductive Technology Versus Spontaneous Conception: A Systematic Review and Meta-Analysis
Source: Diagnostics (Basel). 2025 Aug 29;15(17):2192. doi: 10.3390/diagnostics15172192 (PMC12427646; doi:10.3390/diagnostics15172192)
Supplement: Supplementary file 1 [file diagnostics-15-02192-s001.zip › Supplementary Table.pdf]

Supplementary table S1. Excluded studies that may seem to fulfill our criteria

Study, Year	Study period	Study type	Country	Inclusion criteria	Exclusion criteria	Control – study group	UtA Doppler measurements	Time of the measurements	Exclusion reason
Adibi et al, 2015[1]	January 2013 to January 2014	Prospective cohort study	Iran	Age < 40 years, infertile women with frequent abortions, regular menstrual cycle, non-polycystic ovaries at enrollment, FSH < 10 IU/L on day 3.	Tubal factor infertility, history of uterine surgery and/or apparent endometrial pathology, clinically relevant systemic diseases (diabetes, ulcerative colitis, Crohn's disease, connective tissue diseases, hypertension).	Clinical pregnancy – no pregnancy	PI, RI, PSV	Day of hCG injection	Not relevant study and control groups, UtA PI measurement before 1st trimester scan
Cacciatore et al, 1996[2]	-	Prospective observational self-controlled study	Finland	Infertile women in the IVF-ET program at Helsinki University Central Hospital.	-	IVF artificial cycle – IVF natural cycle	PI	During the menstrual cycle and prior to embryo transfer in an IVF cycle.	No SC group, UtA PI measurement before the 1st trimester scan
Cacciatore et al, 1997[3]	-	Prospective observational self-	Finland	Infertile women participating in the IVF-ET program.	-	IVF artificial cycle – IVF natural cycle	PI	During the menstrual cycle and 4 days after hCG	No SC group, UtA PI measurement before the 1st trimester scan

		controlled study						administration in a subsequent gonadotropin-stimulated cycle.	
Chiang, 2000[4]	December 1997 to December 1998	Prospective cohort study	Taiwan	Age ≥ 40 years, normal uterine cavity, IVF-ET.	-	Clinical pregnancy – no pregnancy	PI	During the menstrual cycle	Not relevant study and control groups, UtA PI measurement before 1st trimester scan
Crosby et al, 2022[5]	October 2016 to February 2018	Prospective cohort study	Ireland	Age < 38 years, no previous pregnancy, regular menstrual cycles, no steroid hormone use within three months, normal transvaginal ultrasound.	-	Clinical pregnancy – no pregnancy	PI, RI	During the menstrual cycle	Not relevant study and control groups, UtA PI measurement before 1st trimester scan
Ergin et al, 2015[6]	May 1999 - December 2013	Retrospective cohort study	Turkey	Twin pregnancies without anatomical or genetic fetal anomalies,	Fetuses with no information on spontaneous or IVF conception, and fetuses with undetermined	IVF/ICSI	Mean UtA-PI	Second trimester	No data for singleton pregnancies

				biochemical abnormalities, or familial genetic diseases.	second-trimester uterine artery impedance.				
Geipel et al, 2001[7]	January 1995 - July 1999	Retrospective case-control study	Germany	ICSI patients with uterine artery Doppler studies at 18–24 weeks gestation as part of second-trimester sonogram.	All fetuses with malformations or other indications (suspected abnormality, growth retardation) outside of screening criteria.	IVF/ICSI	RI	18-24 gestational weeks	No UtA PI data
Fan et al, 2023[8]	April 2021 to Septembe r 2021	Retrospective cohort study	China	Age < 38 years, first FET, voluntary uterine artery Doppler monitoring during the implantation window, infertility due to fallopian tube abnormality or mild oligoasthenospermia.	Preimplantation genetic testing, endometriosis or adenomyosis, polycystic ovarian syndrome, ovarian reserve decrease, ovulation disorders, uterine malformations, severe oligospermia/azoospermia/necrozoospermia, $\ge$2 pregnancy losses, serious medical diseases.	Clinical pregnancy – no pregnancy	PI, RI, PSV	Day of ET	Not relevant study and control groups, UtA PI measurement before 1st trimester scan

Haapsamo et al, 2009[9]	-	Randomized double-blind study	Finland	Less than four previous ovarian stimulations and no contraindications for aspirin.	-	Clinical pregnancy – no pregnancy	PI	Day of ET	Not relevant study and control groups, UtA PI measurement before 1st trimester scan
Hoozemans et al, 2008[10]	March 2002 to December 2003	Prospective cohort study	Netherlands	Age < 39 years, regular menstrual cycle, two normal (non-polycystic) ovaries, FSH < 10 IU/L on day 3.	Tubal factor infertility, history of uterine surgery/endometrial pathology, clinically relevant systemic diseases, smoking, BMI > 28 kg/m².	Clinical pregnancy – no pregnancy	PI	Day of hCG injection, day of ET	Not relevant study and control groups, UtA PI measurement before 1st trimester scan
Khan et al, 2016[11]	June 2011 to April 2014	Prospective cohort study	India	Age 24–43 years, infertility duration 2–20 years.	Patients with secondary infertility and patients with associated male subfertility and infertility.	Clinical pregnancy – no pregnancy	PI	During the menstrual cycle	Not relevant study and control groups, UtA PI measurement before 1st trimester scan
Li et al, 2024[12]	October 2019 to Septembe r 2020	Retrospective cohort study	China	Age < 40 years, women undergoing FET.	Chromosomal abnormalities, congenital uterine dysplasia, intrauterine adhesions, hydrosalpinx, comorbid medical diseases (hypertension, diabetes, thyroid dysfunction, liver dysfunction,	Clinical pregnancy – no pregnancy	PI	Day of endometrial transformation before ET	Not relevant study and control groups, UtA PI measurement before 1st trimester scan

					thrombocytopenia), acute hemorrhagic diseases (peptic ulcer), missing cycle data.				
Ng et al, 2004[13]	Between July 2002 and November 2003	Prospective observational self-controlled study	China	Infertile patients attending the Assisted Reproduction Unit at the University of Hong Kong for IVF treatment, having undergone both stimulated cycles and subsequent FET.	(i) Distortion of the uterine cavity shown on ultrasound scanning and (ii) use of clomiphene citrate or HRT in FET cycles.	IVF artificial cycle – IVF natural cycle	PI	On the day of hCG injection or during the natural menstrual cycle	No SC group, UtA PI measurement before the 1st trimester scan
Salle et al,1998[14]	1996	Prospective cohort study	France	Women undergoing IVF, normal serum FSH on day 3 of the cycle.	Older than 38 years old.	Clinical pregnancy – no pregnancy	PI	During the menstrual cycle	Not relevant study and control groups, UtA PI measurement before 1st trimester scan
Tayyar et al, 2015[15]	January 2006 to March 2014	Prospective cohort study	UK	Singleton pregnancy delivering a phenotypically normal live birth or stillbirth at or after 24 weeks' gestation.	Pregnancies with aneuploidies or major fetal abnormalities and those ending in termination, miscarriage or fetal death before 24 weeks.	IVF and ovulation induction / intrauterine insemination –	Multivariable model with UtA PI	1st, 2nd and 3rd trimester	No reporting of UtA-PI values or relevant effect measures for the groups of interest

						spontaneous conception			
Zaidi et al, 1996[16]	1994	Prospective cohort study	London	Infertile women undergoing IVF.	-	Clinical pregnancy – no pregnancy	PI	Day of hCG injection	Not relevant study and control groups, UtA PI measurement before 1st trimester scan
Zollner et al, 2012[17]	-	Prospective cohort study	Germany	Couples were included only if at least one fertilized oocyte could be transferred.	Women with fibroids of the uterus or with uterine abnormalities were excluded.	Clinical pregnancy – no pregnancy	PI, RI, PSV	Day of ET	Not relevant study and control groups, UtA PI measurement before 1st trimester scan

Abbreviations: ART, Assisted Reproductive Technology; BMI, Body Mass Index; ET, Embryo Transfer; FSH, Follicle Stimulating Hormone; hCG, Human Chorionic Gonadotropin; HRT, Hormone Replacement Therapy; ICSI, Intracytoplasmic Sperm Injection; IVF, In Vitro Fertilization; MoM, Multiple of Median; PI, Pulsatility Index; PSV, Peak Systolic Velocity; RI, Resistivity Index; SC, spontaneous conception; UtA, Uterine Artery

References

1. Adibi, A.; Khadem, M.; Mardanian, F.; Hovsepian, S. Uterine and arcuate arteries blood flow for predicting of ongoing pregnancy in in vitro fertilization. *Journal of research in medical sciences : the official journal of Isfahan University of Medical Sciences* **2015**, *20*, 879–884, doi:10.4103/1735-1995.170622.
2. Cacciatore, B.; Simberg, N.; Fusaro, P.; Tiitinen, A. Transvaginal Doppler study of uterine artery blood flow in in vitro fertilization-embryo transfer cycles. *Fertility and sterility* **1996**, *66*, 130–134, doi:10.1016/s0015-0282(16)58400-3.
3. Cacciatore, B.; Simberg, N.; Tiitinen, A.; Ylikorkala, O. Evidence of interplay between plasma endothelin-1 and 17 beta-estradiol in regulation of uterine blood flow and endometrial growth in infertile women. *Fertil Steril* **1997**, *67*, 883–888, doi:10.1016/s0015-0282(97)81401-x.
4. Chiang, C.H.; Hsieh, T.T.; Chang, M.Y.; Shiau, C.S.; Hou, H.C.; Hsu, J.J.; Soong, Y.K. Prediction of pregnancy rate of in vitro fertilization and embryo transfer in women aged 40 and over with basal uterine artery pulsatility index. *Journal of assisted reproduction and genetics* **2000**, *17*, 409–414, doi:10.1023/a:1009405000032.
5. Crosby, D.A.; Glover, L.E.; Downey, P.; Mooney, E.E.; McAuliffe, F.M.; O'Farrelly, C.; Brennan, D.J.; Wingfield, M. Mid-luteal uterine artery Doppler indices in the prediction of pregnancy outcome in nulliparous women undergoing assisted reproduction. *Human fertility (Cambridge, England)* **2022**, *25*, 670–676, doi:10.1080/14647273.2021.1872111.
6. Ergin, R.N.; Yayla, M. Comparison of second trimester uterine artery pulsatility index between IVF and spontaneous twin pregnancies. *Clin Exp Obstet Gynecol* **2015**, *42*, 614–616.
7. Geipel, A.; Ludwig, M.; Germer, U.; Katalinic, A.; Diedrich, K.; Gembruch, U. Uterine artery Doppler velocimetry and the outcome of pregnancies resulting from ICSI. *Hum Reprod* **2001**, *16*, 1397–1402, doi:10.1093/humrep/16.7.1397.
8. Fan, J.; Zhang, J.; Xu, S.; Liu, H.; Lv, W.; Bi, X.; Liu, Y.; Shi, W.; Zhang, Y.; Wu, X. The predictive value of uterine artery Doppler in the success rate of pregnancy from the first frozen embryo transfer during the implantation window. *BMC pregnancy and childbirth* **2023**, *23*, 825, doi:10.1186/s12884-023-06150-y.
9. Haapsamo, M.; Martikainen, H.; Räsänen, J. Low-dose aspirin and uterine haemodynamics on the day of embryo transfer in women undergoing IVF/ICSI: a randomized, placebo-controlled, double-blind study. *Human reproduction (Oxford, England)* **2009**, *24*, 861–866, doi:10.1093/humrep/den489.
10. Hoozemans, D.A.; Schats, R.; Lambalk, N.B.; Homburg, R.; Hompes, P.G. Serial uterine artery Doppler velocity parameters and human uterine receptivity in IVF/ICSI cycles. *Ultrasound in obstetrics & gynecology : the official journal of the International Society of Ultrasound in Obstetrics and Gynecology* **2008**, *31*, 432–438, doi:10.1002/uog.5179.
11. Khan, M.S.; Shaikh, A.; Ratnani, R. Ultrasonography and Doppler Study to Predict Uterine Receptivity in Infertile Patients Undergoing Embryo Transfer. *Journal of obstetrics and gynaecology of India* **2016**, *66*, 377–382, doi:10.1007/s13224-015-0742-5.

12. Li, L.; Du, M.; Wu, S.; Wen, C.; Kong, P.; Zhang, J.; Guan, Y. Analysis of the uterine artery pulsatility index on the day of endometrial transformation and pregnancy outcomes of patients undergoing frozen-thawed embryo transfer. *Frontiers in endocrinology* **2024**, *15*, 1278504, doi:10.3389/fendo.2024.1278504.
13. Ng, E.H.; Chan, C.C.; Tang, O.S.; Yeung, W.S.; Ho, P.C. Comparison of endometrial and subendometrial blood flow measured by three-dimensional power Doppler ultrasound between stimulated and natural cycles in the same patients. *Hum Reprod* **2004**, *19*, 2385–2390, doi:10.1093/humrep/deh384.
14. Salle, B.; Bied-Damon, V.; Benchaib, M.; Desperes, S.; Gaucherand, P.; Rudigoz, R.C. Preliminary report of an ultrasonography and colour Doppler uterine score to predict uterine receptivity in an in-vitro fertilization programme. *Human reproduction (Oxford, England)* **1998**, *13*, 1669–1673, doi:10.1093/humrep/13.6.1669.
15. Tayyar, A.; Guerra, L.; Wright, A.; Wright, D.; Nicolaides, K.H. Uterine artery pulsatility index in the three trimesters of pregnancy: effects of maternal characteristics and medical history. *Ultrasound Obstet Gynecol* **2015**, *45*, 689–697, doi:10.1002/uog.14789.
16. Zaidi, J.; Pittrof, R.; Shaker, A.; Kyei-Mensah, A.; Campbell, S.; Tan, S.L. Assessment of uterine artery blood flow on the day of human chorionic gonadotropin administration by transvaginal color Doppler ultrasound in an in vitro fertilization program. *Fertility and sterility* **1996**, *65*, 377–381, doi:10.1016/s0015-0282(16)58103-5.
17. Zollner, U.; Speckteter, M.-T.; Zollner, K.-P.; Dietl, J. Uterine artery blood flow in the periimplantation period in embryo transfer cycles. *Asian Pacific Journal of Reproduction* **2012**, *1*, 177–182, doi:https://doi.org/10.1016/S2305-0500(13)60073-1.
